# Emerging antibiotic resistance by various novel proteins/enzymes

**DOI:** 10.1007/s10096-025-05126-4

**Published:** 2025-04-15

**Authors:** Shengwei Sun

**Affiliations:** 1 School of Engineering Sciences in Chemistry, Biotechnology and Health, Department of Fibre and Polymer Technology, KTH Royal Institute of Technology, Stockholm, SE-100 44 Sweden; 2 School of Engineering Sciences in Chemistry, Biotechnology and Health, Science for Life Laboratory, Tomtebodavägen 23, Solna, 171 65 Sweden

**Keywords:** Antibiotics, Antibiotic resistance, Resistance mechanisms, Proteins/enzymes, Combating strategies

## Abstract

**Background:**

The emergence and dissemination of antibiotic resistance represents a significant and ever-increasing global threat to human, animal, and environmental health. The explosive proliferation of resistance has ultimately been seen in all clinically used antibiotics. Infections caused by antibiotic-resistant bacteria have been associated with an estimated 4,950,000 deaths annually, with extremely limited therapeutic options and only a few new antibiotics under development. To combat this silent pandemic, a better understanding of the molecular mechanisms of antibiotic resistance is immensely needed, which not only helps to improve the efficacy of current drugs in clinical use but also design new antimicrobial agents that are less susceptible to resistance.

**Results:**

The past few years have witnessed a number of new advances in revealing the molecular mechanisms of AMR. Following five sophisticated mechanisms (efflux pump, antibiotics inactivation by enzymes, alteration of membrane permeability, target modification, and target protection), the roles of various novel proteins/enzymes in the acquisition of antibiotic resistance are constantly being described. They are widely used by clinical bacterial strains, playing a key role in the emergence of resistance.

**Conclusion:**

While most of these have so far received less attention, expanding our understanding of these emerging resistance mechanisms is of crucial importance to combat the antibiotic resistance crisis in the world. This review summarizes recent advances in our knowledge of emerging resistance mechanisms in bacteria, providing an update on the current antibiotic resistance threats and encouraging researchers to develop critical strategies for overcoming the resistance.

## Introduction

Antibiotics are a class of compounds initially used for the treatment and prevention of bacterial infections. Unfortunately, with the overuse and misuse of antibiotics, bacteria begin to develop the ability to defeat them, leading to the emergence of antimicrobial resistance (AMR) and antibiotic-resistant bacteria (ARB) [1–3]. At present, the problem of AMR is becoming increasingly serious worldwide and has become one of the major causes of disease and death, causing a serious global health crisis. According to statistics by *The Lancet*, antibiotic-resistant infections killed 1.27 million people in 2019 alone [4], and are responsible for roughly 5 million deaths in 2022 [5]. It is estimated that by 2050, this number will increase to 10 million people per year, significantly exceeding the number of cancer deaths (8.2 million) [5, 6]. Moreover, AMR also poses a significant burden on healthcare systems, resulting in high global health expenditures. Based on the US Centers for Disease Control and Prevention (CDC) data, antibiotic-resistant infections are estimated to cost up to $20 billion in annual healthcare costs and $35 billion in lost productivity [7]. To combat the growing threat of AMR to human health and the economy, understanding the underlying molecular mechanisms is crucial for developing new strategies to treat infectious diseases.

**Fig. 1:**
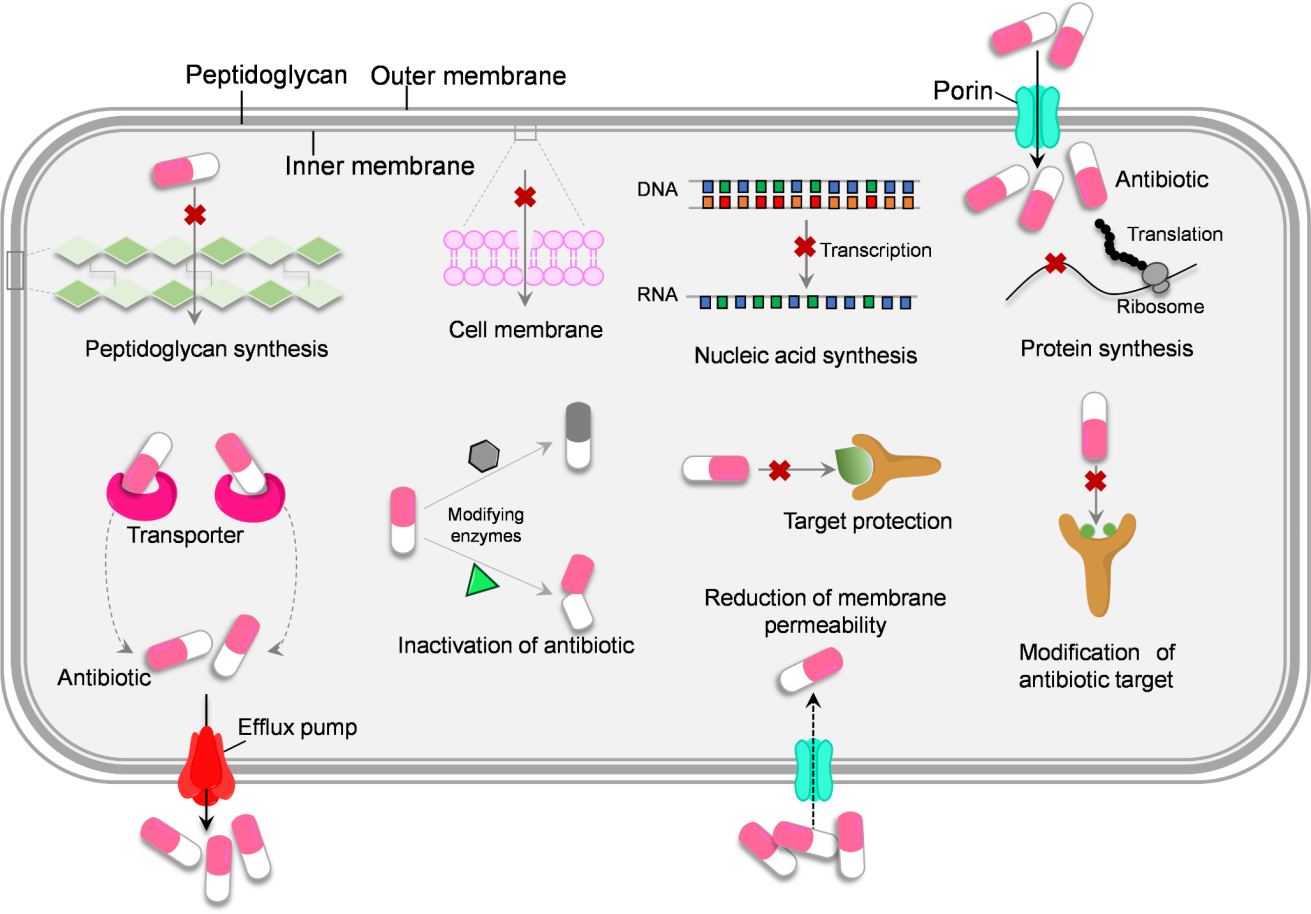
General mechanisms for antibiotic action and antimicrobial resistance. The antibacterial activity of antibiotics relies on four main mechanisms: (1) inhibition of peptidoglycan synthesis; (2) disruption of the cell membrane; (3) inhibition of nucleic acid synthesis; and (4) interfering with protein synthesis [9]. For typical AMR mechanisms, efflux pump, inactivation of antibiotics by modifying enzymes, alteration of membrane permeability, modification of antibiotic target, and target protection are included [11]

An increasing number of studies are revealing the mechanisms of action of antibiotics and the AMR mechanisms in environmental microbes (Fig. 1). The antibiotics usually share the same ecological niche with bacteria, and it is generally believed that growth inhibition is their main ecological function in the environment. There are several essential antibiotic targets in bacteria (e.g., cell wall, cell membrane, nucleic acid, and ribosome) [8, 9]. Accordingly, the antibiotic action mode includes (1) inhibition of peptidoglycan synthesis in the process of forming the cell wall; (2) interfering with cell membrane function through the eventual disruption of the outer and cytoplasmic membrane; (3) inhibition of nucleic acid synthesis through inhibiting bacterial RNA polymerase activity and blocking transcription; (4) targeting protein synthesis. Unfortunately, in order to survive, bacteria retain an outstanding capacity for rapid adaptation and evolution in response to various antibiotics through various molecular mechanisms [10].

Bacteria can be intrinsically resistant to one or more classes of antibiotics. They can also acquire such resistance by either (1) genetic mutations in the chromosome or (2) acquired exogenous DNA via horizontal gene transfer encoding resistance determinants [12]. A number of genes that confer intrinsic resistance to different kinds of antibiotics (such as β-lactams, fluoroquinolones, and aminoglycosides) have been identified in the genomes of bacteria [11, 13]. Mutations are a permanent change for bacteria to become resistant to antibiotics, which typically involve a few types of genes, including those encoding the antibiotic targets, antibiotic transporters, and regulators that modulate the expression of transporters, such as efflux pumps and modifying enzymes, thereby causing AMR [14]. Acquired antibiotic resistance is the result of an evolutionary process where bacteria are endowed with various mechanisms, such as active efflux of the antibiotic, enzymatic modification/degradation of the antibiotic, alteration of membrane permeability, modification of antibiotic targets, and target protection [15, 16]. These sophisticated mechanisms have been well-documented in previous studies [11, 17–19] and significantly contributed to the development and clinical use of many AMR breakers, such as antibiotic resistance gene (ARG) silencers, ribosomal inhibitors, and efflux pump inhibitors [20]. However, AMR is a naturally occurring process that will never go away [21, 22]. Bacteria, especially those prevalent pathogens (e.g., methicillin-resistant *Staphylococcus aureus*, penicillin and β-lactam-resistant *Streptococcus pneumoniae*, pan-resistant *Acinetobacter baumannii*, and multidrug-resistant *Pseudomonas aeruginosa*) are always evolving new ways to avoid the effects of the antibiotic [23]. Hence, the continued fight against bacterial infections/resistance to antibiotics is required.

Over the past years, new technological advances have driven continuous progress in revealing the molecular mechanisms of AMR. The underlying resistance mechanisms contribute to our understanding of the evolution of AMR in bacteria. More importantly, clarifying these mechanisms is crucial for improving antibiotic efficacy, obtaining answers, and exploring options to combat bacterial resistance to preserve the utility of antibiotics for years to come. In these cases, following the five general mechanisms (Fig. 1), this review summarizes recent progress in understanding AMR by compiling emerging evidence from previous studies (2020–2025), which may offer insights into the development of a novel approach to overcome AMR in environmental and clinical settings.

### Efflux pump-like proteins

Efflux pumps play a prominent role in controlling the accumulation and transportation of antibiotics in resistant bacteria. It is the most studied and targeted AMR mechanism. The first efflux pump expelling tetracycline in *Escherichia coli* was discovered in 1980 [24]. Since then, various efflux mechanisms have been described to account for bacterial infections. According to the substrate selectivity, protein structure, and energy source, several well-known antibiotic efflux pump families have been identified, including the major facilitator superfamily (MFS), the ATP-binding cassette (ABC) superfamily, the drug metabolite transporter (DMT) superfamily, the small multidrug resistance (SMR) family, the resistance-nodulation-division (RND) superfamily, the multidrug and toxic compound extrusion (MATE) family, and the proteobacterial antimicrobial compound efflux (PACE) superfamily [25]. Most plasmid-carried or chromosomal genes encoding efflux pumps among gram-positive bacteria belong to the MFS and ABC families. In contrast, the predominant clinically relevant efflux system in gram-negative bacteria usually comes from a member of the RND superfamily, comprising an inner-membrane protein, periplasmic adapter proteins, and outer-membrane protein. Accumulating evidence demonstrates that the efflux pumps are widely implicated in (a) efflux of antibiotics, (b) regulation of host physiology, (c) biofilm formation, (d) metal resistance, and (e) virulence [26], suggesting they are more clinically important than usually thought. Recently, there has been an increasing number of proteins with functions similar to efflux pumps. These efflux pump-like proteins have undeniable significance in intrinsic and acquired resistance to various antibiotics in bacteria. Some reported examples are shown below.

#### BON domain-containing protein

**Fig. 2:**
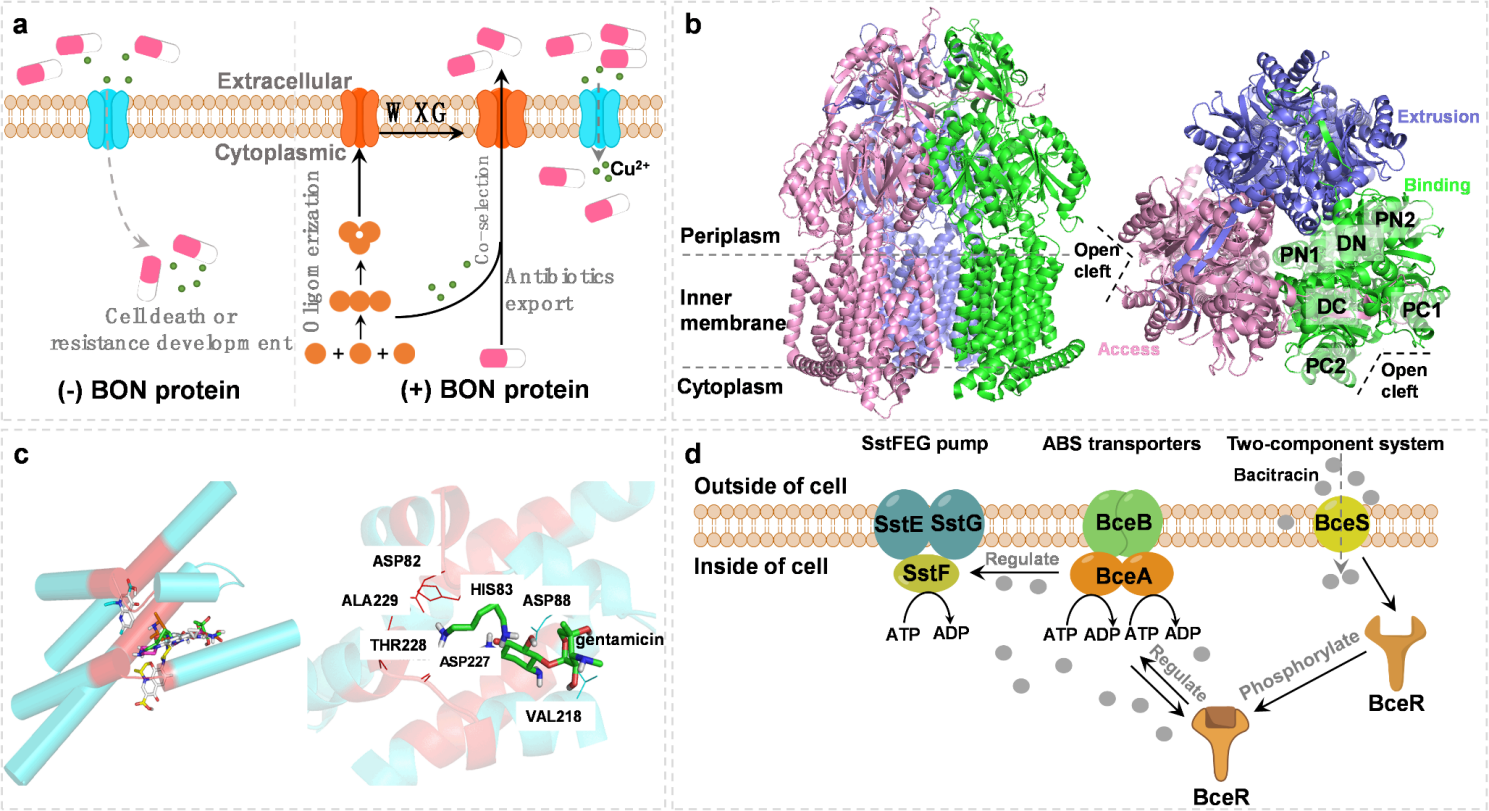
Emerging efflux pump-like proteins-induced antibiotic resistance mechanisms. (**a**) BON domain-containing protein-mediated co-selection of antibiotic and heavy metal resistance in bacteria [30]. (**b**) Cryo-electron microscopy structures of a *Campylobacter* multidrug efflux pump CmeABC without antibiotics [31]. Each subunit of CmeB is labelled with a different colour. The six subdomains DN, DC, PN1, PN2, PC1, and PC2 are labelled. (**c**) A NiCoT family metal transporter from *Mycobacterium tuberculosis* (Rv2856/NicT) behaves as a drug efflux pump facilitating cross-resistance to antibiotics [32]. All the antibiotics in the active site are shown after docking. The docking interaction of NicT with the gentamicin on the right side is used as an example. Several key amino acid residues are labelled. (**d**) Bacitracin resistance and enhanced virulence of *Streptococcus suis* via a novel membrane transporter SstFEG [33]

Efflux pumps have been identified as the dominant resistance mechanism in nearly all organisms, while other cytoplasmic and membrane proteins have received little attention. Nevertheless, they play an equally important role in promoting resistance to infections. These intrinsic resistances in many bacteria represent even more serious therapeutic problems. Among them, the BON (bacterial OsmY and nodulation), a putative membrane-binding domain, is one of the most abundant proteins in the cell membrane [27]. It has been demonstrated to bind noncovalently to peptidoglycan and chitin through the interaction with N-acetyl glucosamine moieties [28]. A recent study has suggested that the BON domain-containing protein exhibits a high affinity toward carbapenem antibiotics [29]. The bacteria containing BON protein showed fold increases in higher minimum inhibitory concentration (MIC) values of imipenem and meropenem in comparison to the NP-6 clinical strain and *E. coli* DH5α. Molecular dynamic simulation studies revealed that BON protein can form stable complexes with meropenem and imipenem. Subsequently, Sun et al. reported another new type of BON domain-containing protein with an efflux pump-like function which was identified from the soil metagenomic libraries [30]. It helped bacteria resist diverse antibiotics, with the highest efflux activity toward ceftazidime. A more than 32-fold increase in MIC was observed. Moreover, the BON protein was active in the co-selection of antibiotic and metal ion resistance in the bacterial cells. Structurally, the BON protein can undergo self-assembly into a trimer complex, forming a pore-shaped channel to transport antibiotics. A conservative WXG motif in the BON protein is essential for the substrate-transporting function, formation of oligomeric pores, and interactions between proteins and cell membranes. Based on these findings, an AMR mechanism, namely “one-in, one-out”, was proposed for the first time (Fig. 2a). Further studies are required to shed light on the sequence-structure-function relationships in the BON domain-containing protein. For example, exploring the sequence and structural diversity, determining the crystal structure, and understanding the resistance mechanism would fill the knowledge gap of BON domain-containing protein-mediated antibiotic resistance.

#### CmeABC multidrug efflux system

CmeABC multidrug efflux system consists of three tripartite protein components, including CmeA, CmeB, and CmeC, which play a central role in mediating multidrug resistance to many antibiotics such as chloramphenicols, fluoroquinolones, tetracycline, and macrolides in *Campylobacter jejuni* (*C. jejuni*) [34]. CmeB contains the specific substrate binding sites involved in the proton-relay network that are responsible for the proton motive force-dependent active transport. CmeA is a periplasmic membrane fusion protein, while CmeC forms an outer membrane channel. Three components play a synergistic role in antibiotic expulsion [35]. Recently, Yao et al. identified a potent variant of CmeABC, namely RE-CmeABC, which significantly enhanced Campylobacter’s resistance to multiple antibiotics [36]. It was found that the *C. jejuni* strain carrying the RE-CmeABC expanded mutational selectivity to ciprofloxacin, increased the frequency of mutation to ciprofloxacin resistance, and altered the MIC distributions of several drugs in clinical *C. jejuni* isolates to a higher range. Consequently, the RE-CmeABC efflux system is considered an effective strategy utilized by *Campylobacter* for adaptation to antibiotic selection, representing an emerging multidrug-resistant mechanism [36]. To elucidate the molecular mechanisms underlying antibiotic resistance, Zhang et al. determined the 3D structures of this membrane protein RE-CmeB in complex with some antibiotics such as chloramphenicol, ciprofloxacin, ampicillin, and erythromycin [31] (Fig. 2b). Based on these 3D structures, key ligand-binding residues as well as important interactions between RE-CmeB and antibiotics were identified. It suggested that different subsets of amino acid residues in RE-CmeB protein were involved in binding the antibiotics, which contributes to the optimization of the substrate identification patterns and a strong capability to extrude a broad spectrum of antibiotics effectively. Further structural investigations are needed to explain the detailed molecular mechanisms of RE-CmeB transporter controlling multidrug recognition. How RE-CmeB transports antibiotics out of cells and which amino acid residues play important roles in this process are unanswered scientific questions.

#### NiCoT transporter

Metal ions often serve as co-selecting factors in the proliferation of AMR in human pathogens from given environmental settings. The resulting enhanced antibiotic transport typically results in low levels of intrinsic susceptibility, acquisition of collateral resistance mechanisms, and cross-resistance to chemically unrelated compounds. For example, the NiCoT transporter family is one of the most widespread Ni/Co transporters in various organisms [37]. Recently, Adhikary et al. reported the ability of a putative NiCoT family transporter, Rv2856 or NicT from *Mycobacterium tuberculosis* (Mtb), to transport nickel/cobalt and antibiotics and identified a group of key amino acid residues responsible for its function [32]. The increase of NicT-induced intracellular nickel uptake in *E. coli* CS109 and *Mycobacterium smegmatis* resulted in enhanced resistance to several antibiotics such as ofloxacin, gentamicin, norfloxacin, sparfloxacin, isoniazid and nalidixic acid. Compared with cells without NicT, intracellular accumulation of norfloxacin was relatively low when NicT was expressed in the bacterial cells, suggesting that NicT is involved in the active process of antibiotic efflux. Furthermore, results of the docking study revealed that a number of conserved residues such as Asp82, His83, and Asp88 in domain II of NicT contributed to the formation of the H-bond networks (Fig. 2c). The residues Ala81, Ile84, His112, Phe222, Asp227, Thr228 and Thr230 were the major contributors to hydrophobic and van der Waals interactions. The study demonstrated that Rv2856/NicT was able to actively export different antibiotics and yield cross-resistance to certain antibiotics in the presence of nickel.

#### AadT pump

In clinical settings, multidrug efflux pumps have been described in the development of *Acinetobacter* strains’ resistance to multiple antibiotics [38]. The genes encoding putative pumps are mostly identified in the core genome of the species [39]. Others are implicated with the concerted activities of mobile genetic elements (e.g., transposons and plasmids), dispersed in a subset of phylogenetically distant bacterial strains [40]. For instance, the tetracycline efflux pump, TetB, which was initially discovered on the transposon Tn10 in *E. coli*, was reported in many *Acinetobacter* strains and other gram-negative species [41]. Recently, a group of genes encoding an efflux pump belonging to the Drug: H + antiporter 2 (DHA2) family were identified in several plasmids of *Acinetobacter* [42]. The novel DHA2 family pump proteins can reduce susceptibility to a variety of antibiotics. Moreover, the genes encoding pump homologs are prevalent but invariably related to variants at the AdeAB(C) locus in the corresponding *Acinetobacter* species. Among them, AadT, a new efflux pump protein, was found in the plasmids of *Acinetobacter*’s multidrug resistance [43]. It showed a strong ability to reduce bacterial susceptibility to several antimicrobials, such as erythromycin, tetracycline, and chlorhexidine. The AadT homologs are usually adjacent to the AdeAB(C) efflux pump variants in many *Acinetobacter* species, suggesting that AadT might be able to cooperate with AdeAB(C) variants in the *Acinetobacter* resistance arsenal. More research data is required to confirm the relationships between AddT and other efflux pumps in the *Acinetobacter* species.

#### Novel membrane transporter-SstFEG

*Streptococcus suis* is among the top zoonotic pathogens that cause diseases in pigs and humans, representing a global health problem in the swine industry [44]. It is widely present in the breeding environment of pigs and is considered a reservoir for antibiotic resistance genes [45, 46]. Bacitracin is a polypeptide antibiotic used extensively as a growth promoter in animal husbandry [47]. However, long-time use of bacitracin leads to an increase in resistance genes in microbial strains. Several bacitracin resistance mechanisms have been uncovered over the last decade [48, 49]. For example, the Bce systems composed of the regulator BceSR and the transporter BceAB are widely distributed in bacteria and have been identified as an efflux pump conferring resistance to bacitracin [50]. More recently, a study showed that a potential efflux pump SstFEG is located upstream of the known bacitracin resistance genes *bceAB* and *bceRS* [33] (Fig. 2d). The deletion of *sstFEG* significantly decreased the mutant susceptibility to bacitracin in comparison to wildtype *S. suis* strain. Furthermore, it was found that both the BceAB transporter and the two-component system BceRS were required for SstFEG-mediated bacitracin resistance. Additionally, by dissecting the competitive survival advantage of *S. suis* in animal infection, SstFEG played an important role in colonization and virulence.

#### SA09310 protein

*Staphylococcus aureus* is a gram-positive pathogen causing a range of clinical diseases [51]. *S. aureus* is a notorious human pathogen partly due to its capacity to survive and fight against a variety of antimicrobial compounds [52]. *S. aureus* has evolved various antibiotic resistance mechanisms in the treatment [53, 54]. Among them, efflux pump-mediated resistances in *S. aureus* have been the most extensively studied [55]. According to in silico analysis, 31 multidrug efflux pumps in the *S. aureus* chromosome cover almost all the pump protein families. Nevertheless, only one-third of them have been previously characterized, while the biological functions of these efflux pumps remain poorly understood [56]. In a recent study, a novel multidrug resistance efflux protein, namely SA09310, encoded in the chromosomes of *S. aureus*, was identified and characterized [57], which consisted of 12 transmembrane helices. A *sa09310* gene knockout mutant (Δ*sa09310*) showed enhanced sensitivity to doxycycline and tetracycline, with the MICs increased by 8-fold and 64-fold, respectively. It was demonstrated that the SA09310 possessed the strong capacity to export tetracycline from intracellular to extracellular space. The high conservation of SA09310 homologs in *Staphylococcus* suggests that protein clusters similar to the SA09310 have general functions. More studies are required to decode the regulatory mechanism of AMR caused by SA09310.

### Inactivation of antibiotics

Some bacteria acquire AMR by enzymatically degrading or modifying antibiotic molecules to render them inactive and potentially protect neighboring susceptible cells from antibiotic exposure, which is increasingly prevalent among pathogens. For example, the β-lactamases are a well-known example of antibiotic degradation enzymes, which are mostly produced by the enterobacterales and some species of gram-positive bacteria (e.g., *Enterococcus faecalis*, *S. aureus*, and *Enterococcus faecium* [58]. Based on the protein sequences, they can be separated into four distinct classes (A, B, C, and D) and are highly efficient in inactivating commonly used β-lactam antibiotics by breaking the β-lactam ring open, making them ineffective in binding with the target [59]. In addition, the transfer of chemical groups such as phosphoryl, acetyl, and adenyl is the most common pathway of antibiotic inactivation. A number of transferases (such as *N*-acetyl transferases, *O*-phosphotransferases, and *O*-adenyltransferases) have been identified and characterized for their ability to transfer these chemical groups. Phosphorylation and adenylation are the primary approaches rendering aminoglycosides inactive, while acetylation is widely utilized to inactivate streptogramins, fluoroquinolones, and chloramphenicol [15]. More recently, several studies have reported that oxidative and reductive inactivation of antibiotics often leads to novel resistance mechanisms. Expanding our understanding of these emerging AMR mechanisms is of crucial importance to combat the AMR crisis in the world.

#### Oxidative inactivation

**Fig. 3:**
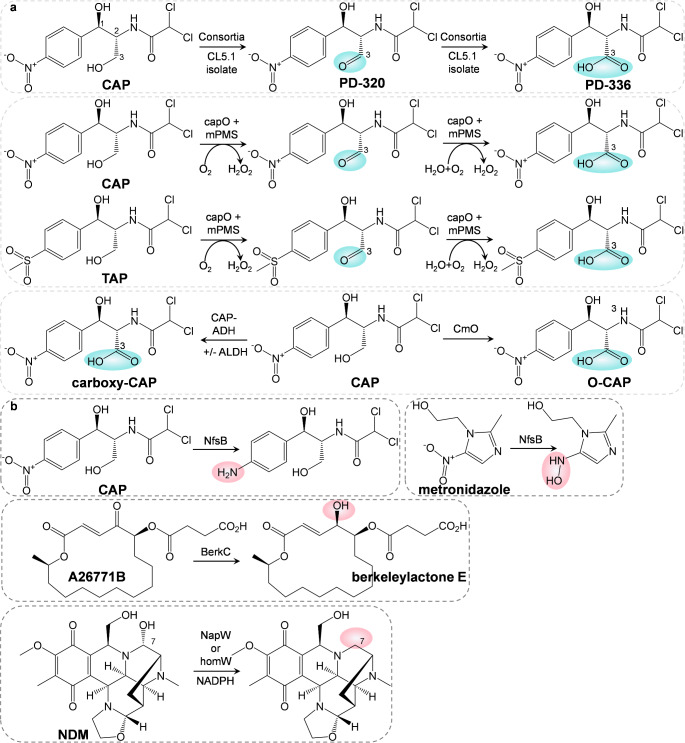
Oxidative and reductive inactivation of antibiotics. (**a**) Oxidative inactivation of antibiotics, such as CAP and TAP, by consortia/CL5.1 isolate, capO + mPMS, CAP-ADH +/- ALDH, and CmO, respectively. The oxidized groups are labelled blue. (**b**) Reductive inactivation of antibiotics, including CAP, metronidazole, A26771B, and NDM, by enzymes NfsB, BerkC, NapW, or homW, respectively. The reduced groups are marked with pink colour

Chloramphenicols have become the main focus of research because of the absence of new antibiotic formulations and the emergence of AMR resulting from the overuse and misuse of existing antibiotics. Chloramphenicol (CAP), thiamphenicol (TAP), and florfenicol (FF) are synthetic antibiotics commonly used in the treatment of infections due to their broad range of activity against bacteria and low cost. CAP contains a p-nitrophenyl group, a dichloromethyl moiety, and a propanediol side chain [60]. The accumulation and distribution of chloramphenicols have been recorded in hot spots such as pharmaceuticals, livestock, poultry, and hospital wastewater. They are the main reasons for the development of chloramphenicols-resistant bacteria. Several chloramphenicols resistance mechanisms have been reported, such as enzymatic inactivation [11]. For example, the acetyltransferases-catalyzed acetylation of the propanediol group of CAP and TAP has been frequently recognized in many bacteria [61]. The hydrolysis of amide bonds by hydrolase estDL136 has also been connected to the fast inactivation of CAP, TAP, and FF [62]. In a previous study, Zhang et al. showed a CAP-resistant strain *Sphingomonas* sp. CL5.1 possessed the ability to oxidize the propanediol C3-OH groups of CAP and TAP into carboxyl groups [63] (Fig. 3a), which is a necessary pharmacophore for the CAP and TAP antibacterial activities [64]. This finding represents a novel CAP resistance mechanism in bacterial cells. Subsequently, Zhang et al. discovered a novel oxidoreductase named CapO featuring the oxidative inactivation of CAP and TAP [65]. Ma et al. also reported a novel oxidase gene, *cmO*, identified from *Sphingomonadaceae* [66]. The corresponding oxidase CmO can catalyze the oxidation at the C-1’ and C-3’ positions of CAP and TAP in *Sphingobium* sp. strain. Docking studies showed that CAP was located within the active pocket of CmO through hydrogen-bonding interaction at key amino acid residues, such as G99, Y380, M474, and N518. Zhang et al. further reported the discovery and characterization of an oxidoreductase that inactivates CAP through dual oxidation of the C3 hydroxyl group [67]. Moreover, oxidation of CAP either relies on glucose-methanol-choline-type flavoenzymes alone, or on extra aldehyde dehydrogenases for increased efficiency. Overall, these results significantly expand our knowledge boundary about chloramphenicols resistance mechanisms. Additionally, resistance to rifamycin antibiotics known for their use in treating tuberculosis, has been revealed ranging from primary target modification and antibiotic inactivation to cytoplasmic exclusion [68]. Among them, rifamycin monooxygenases (Rox) are present in a variety of environmental bacteria and have been associated with the decomposition of the antibiotic. For example, Heba et al. showed that the recombinant rifampicin monooxygenase (RifMO) was able to catalyze the incorporation of a single oxygen atom forming an unstable intermediate that eventually is converted to 2′-N-hydroxy-4-oxo-Rif, leading to the rifampicin resistance in *Nocardia farcinica* [68]. Kalinka et al. reported an unprecedented mechanism of rifamycin inactivation initiated by monooxygenation of the 2-position of the naphthyl group, which subsequently resulted in ring opening and linearization of the antibiotic [69]. This represents a unique mechanism of enzymatic inactivation underpinning the broad spectrum of rifamycin resistance.

#### Reductive inactivation

Antibiotics are biologically active because their structures contain highly sensitive and reactive pharmacophores. As such, the organisms must evolve an effective integration strategy of antibiotic action and production for self-protection machinery. From reported evidence of self-resistance, bacteria extensively use enzymes to catalyze the inactivation of active pharmacophores in resistant bacterial pathogens or antibiotic-producing microbes. The β-lactamase, the enzymes that hydrolyze and inactivate β-lactam antibiotics, is a well-known example [70]. Moreover, different cyclopropane hydrolase families were characterized to confer self-resistance engaging in the biosynthesis of colibactin and yatakemycin [71, 72]. Therefore, continued elucidation of different types of self-resistance mechanisms together with antibiotic biosynthesis pathways will contribute to our understanding of enzymatic antibiotic inactivation.

Crofts et al. identified a nitroreductase NfsB in *Haemophilus influenzae* that conferred CAP resistance through nitroreduction [73]. The enzymatic product from chloramphenicol was demonstrated to be amino-chloramphenicol (Fig. 3b). Moreover, the overexpression of the *nfsB* gene in *E. coli* was associated with a significant increase in susceptibility to metronidazole. It was found that metronidazole synergistically attenuates chloramphenicol resistance in *H. influenzae*, and it has a weak inhibitory effect on in vitro reduction of chloramphenicol by NfsB. Recently, by identifying and elucidating the antibiotic biosynthetic pathway, Zhang et al. described a distinctive self-resistance mechanism in the fungus *Penicillium egyptiacum*, which conferred resistance to a macrolide antibiotic, namely A26771B [74]. A redox cycle of antibiotic self-resistance and biosynthesis is formed by intracellular reductive inactivation and extracellular oxidative activation activated by a short-chain reductase and a flavin-dependent oxidase, respectively.

Tetrahydroisoquinoline antibiotics such as saframycin S (SFM-S), naphthyridinomycin (NDM), lemonomycin (LMM), and ecteinascidin 743 (ET-743) have been extensively studied owing to their remarkable biological activities such as antibacterial and antitumor [75]. The hemiaminal pharmacophore is an important group for antibiotic and antitumor potential, while it requires an efficient resistance mechanism to counteract the toxicity of producing microorganisms. Zhang et al. found a secreted enzyme NapU with the ability to control the NDM concentration around cells for self-resistance by performing extracellularly oxidative activation and conditionally over-oxidative inactivation of 7 H-NDM, an intermediate of NDM [76]. Wen et al. further suggested enzymes including NapW and homW [77], which belong to a dehydrogenase/reductase family encoded by genes *napW* and *homW* within and without biosynthetic gene cluster, respectively, were responsible for an intracellularly reductive inactivation of NDM. Further studies confirmed that gene expression helped bacteria develop NDM-resistance; Asp165, a critical amino acid residue essential for catalytic reduction of NapW, was identified based on structural analysis.

#### Antibiotic inactivation by erm proteins

Erm proteins can generally be separated into monomethyltransferase (e.g., ErmN) [78], and dimethyltransferases (e.g., ErmC′) [79]. They are involved in the attack of the exocyclic amino groups of putative adenine residues to decrease the affinity of macrolide-lincosamide-streptogramin B (MLSB) antibiotics, thus conferring bacterial resistance [80, 81]. In a recent study, *Bacillus halodurans* C-125 strain harboring the *mphB* (macrolide phosphotransferase), the *erm* (erythromycin resistance methylase), and putative *mef* (macrolide efflux) genes were demonstrated to be resistant to MLSB antibiotics [82]. The Erm protein from *B. halodurans* C-125 shows amino acid sequence identity at 66.2% and 61.2% with ErmD [83] and Erm [84], respectively, and it could be classified as ErmK. The AR profile and the reverse transcriptase analysis demonstrated that the ErmK is a dimethylase. Moreover, *E. coli* cells carrying ErmK displayed considerable resistance to tylosin and erythromycin. Few studies have reported antibiotic inactivation by ErmK, so more underlying mechanisms could be further elucidated using molecular docking and molecular dynamic simulation approaches. Consequently, Erm proteins have great potential to act as plausible targets for developing inhibitors.

### Altered membrane permeability

A complicated network of activities strongly controls bacterial membrane transport in response to external stress. In gram-negative bacteria, the unique cell envelope composed of lipopolysaccharide (LPS), a highly acylated glycolipid, serves as a major permeability barrier against the uptake of numerous chemicals, including antibiotics. Intrinsically reducing the permeability to specific antibiotics leads to the development of AMR in many family members of gram-negative bacteria. For instance, alterations in cell membrane lipids via surface charge, permeability, fluidity, and stability of the bacterial membrane can affect susceptibility to antibiotics [85]. Moreover, changing the permeability through outer-membrane proteins (OMPs), especially porins, can result in acquired AMR. The amount and type of porins have a significant impact on the entry of antibiotics (e.g., tetracyclines, fluoroquinolones, β-lactams, and chloramphenicol) into the bacterial cell, thus lowering the susceptibility of bacteria to the antibiotics [86]. To effectively perform the antibiotic transport function, the porins in gram-negative bacteria have to undergo an “open-close” switch [87]. Besides, mutations generally significantly impact the expression and function of porin, thus also causing acquired AMR. Combined with other existing resistance mechanisms, such as efflux pumps and enzymatic degradation or modification, mutations affecting the porin expression tend to result in higher resistance levels [88]. Alterations in cell wall peptidoglycan and teichoic acid are also associated with antibiotic-resistant bacteria [89]. Over the last decade, the emergence of the computational approach, crystallographic data from electron microscopy, electrophysiology, X-rays, and mass spectrometry has greatly improved our understanding of the pore-forming complex mechanism in the cell membrane. Further understanding and exploring new resistance mechanisms are needed to enrich our knowledge of antibiotic transport through bacterial outer membranes.

**Fig. 4:**
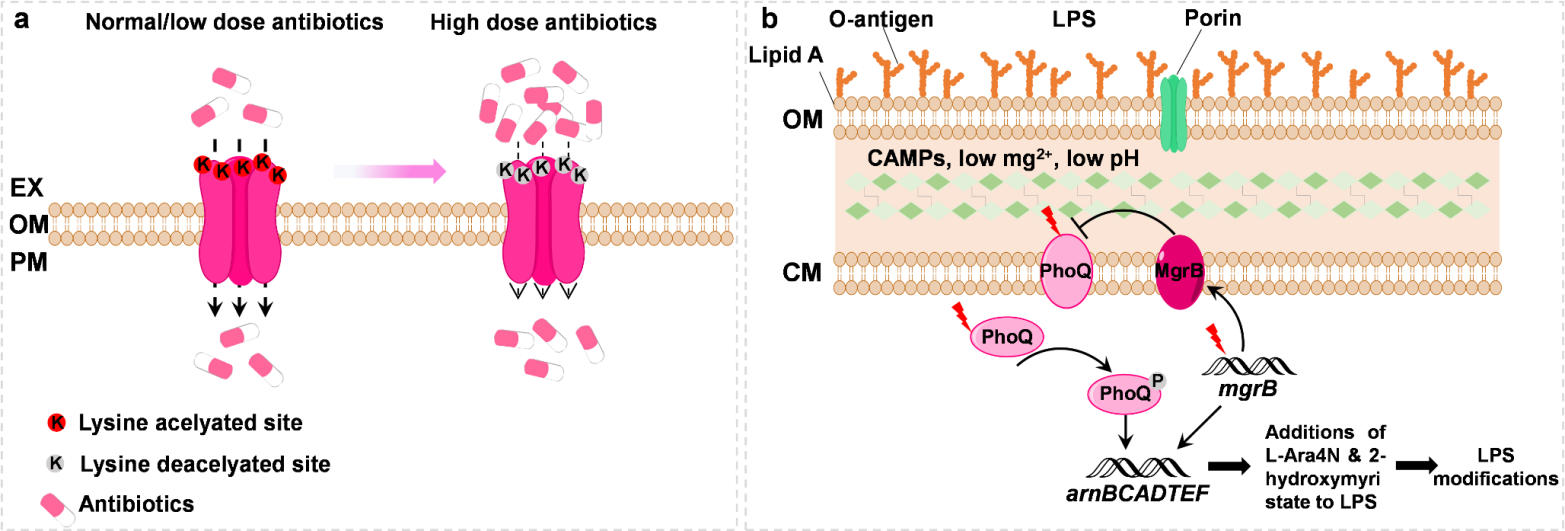
Alteration of membrane permeability. (**a**) The lysine acetylation modification in the porin Aha1 of *Aeromonas hydrophila* regulates the uptake of multidrug antibiotics [94]. EX, OM, and PM indicate extracellular, outer membrane, and periplasmic space, respectively. Kac, lysine acetylation. (**b**) Colistin resistance mechanism associated with MgrB inactivation in *Klebsiella pneumoniae* [95]. OM, outer membrane; CM, cytoplasmic membrane, LPS, lipopolysaccharide, L-Ara4N, 4-amino-4-deoxy-L-arabinose

One of the well-known protein posttranslational modifications (PTMs) is the protein lysine acetylation (Kac) modification, frequently found in eukaryotic and prokaryotic cells. More importantly, it is responsible for various biological functions in bacteria, such as regulating metabolism, quorum sensing, virulence, and chemotaxis [90, 91]. Due to the reversible and dynamic properties of Kac modification, it has been demonstrated to be involved in bacterial AMR. Kac immunoaffinity enrichment and high-resolution mass spectrometry methods have identified Kac modification sites in several AMR proteins. For instance, the subunit B protein of DNA gyrase, namely GyrB, containing Kac modifications on many lysine sites, was reported to confer bacterial resistance to quinolone [90, 92]. Li et al. suggested that 14 AMR proteins with Kac modification differed significantly between the susceptible *Salmonella typhimurium* strains and ciprofloxacin-resistant strains [93]. Recently, Zhang et al. used quantitative proteomics methods to compare the differential expression between oxytetracycline-sensitive (OXYS) and OXY-resistance (OXYR) strains of pathogenic *Aeromonas hydrophila* [94]. It indicated that the expression of an outer membrane protein, namely Aha1, was significantly reduced in both Kac peptide levels and whole protein. The Kac modifications on K57, K187, and K197 sites located at Aha1 had a negative impact on regulating the OXY resistance (Fig. 4a), which was further demonstrated to control OXY entry into the outer membrane. This study shows for the first time that protein Kac can modify outer membrane proteins and directly regulate AMR in bacteria.

Overusing ciprofloxacin (CIP) has caused serious issues in the prevention and treatment of persistent diseases in both developed and developing countries. The development of multi-drug resistance (MDR) in *Salmonella Typhi* is directly related to the outer membrane proteins (OMPs). Recently, Akshay et al. investigated the function of quinolone resistance determining region (QRDR) of *gyrA* and *parC* in CIP-resistant isolates of *S. Typhi* by examining the differential expression of OMPs [96]. Notably, a single point mutation in the *gyrA* gene at codon 83 and a rare amino acid substitution at codon 80 of parC in CIP-resistant isolates were observed. These results offered valuable information on the QRDR mutation and differential expression of OMP-encoding genes between CIP-resistant and susceptible isolates of *S. Typhi*. It is required to further characterize the roles of OMPs and the QRDR region mutation in quinolone-resistant *S. Typhi* isolates.

Similarly, the emergence and spread of colistin resistance is currently confronted with a global health challenge. For example, mutations in the chromosomal gene *mgrB* can increase colistin resistance in *Klebsiella pneumoniae*. The encoding MgrB protein spans the inner membrane and represses PhoP phosphorylation through the inhibition of PhoQ kinase, which is critical for lipid biosynthesis in the bacterial outer membrane (Fig. 4b). Yap et al. recently presented a review to highlight the effect of chromosomal gene *mgrB* mutations on membrane permeability, leading to colistin resistance in *K. pneumoniae* [95]. As the problem of colistin resistance in the world, determining how the *mgrB*-associated membrane changes contributes to new insights and guidance for developing new antibiotics that afford strong membrane penetration ability, thereby possibly addressing the resistance concerns.

In addition, Feng et al. demonstrated that bisphenol S and AF at the environmentally relevant concentrations (0.1–100.0 µg/L) accelerated the occurrence of conjugative transfer frequency of ARGs through the mobilizable plasmid RP4 by 2–5 times within and across bacterial genera [97]. They exhibited no significant effect on bacterial growth but caused a slight alteration of the cell membrane permeability. This bisphenols-induced coupled transfer of ARGs plays a vital role in the dissemination of AMR in the natural environment, wildlife, and human gut microbial communities, becoming one of the most dangerous threats to the global health system.

### Modification of antibiotic target

Introducing modifications to the target site through mutation or post-translational modification has been increasingly adopted by pathogens in the clinic to prevent the antibiotic from binding to the target [98]. This is an effective means of AMR development, usually caused by spontaneous mutations of genes or genes encoding the protein/enzyme as the antibiotic target. For example, many clinically resistant strains often employ chemical modification of the molecular targets in the bacterial cell envelope to develop resistance to polymyxin and glycopeptide antibiotics [99]. Polymyxin resistance occurs through lipopolysaccharide modification, while resistance to glycopeptide is achieved by modifying the D-alanine-D-alanine peptide stem in the bacterial cell wall. Moreover, the presence of alternative penicillin-binding proteins encoded by the *mecA* and *mecC* genes leads to the development of resistant *Staphylococcus* spp. strain with a significantly reduced affinity toward β-lactam antibiotics [100]. Fluoroquinolone resistance is derived from gene mutations that affect the quinolone-resistance-determining region in the DNA gyrase in gram-positive and gram-negative bacteria [101]. The most common changes in *Acinetobacter baumannii* are mutations in the RNA polymerase and DNA gyrase genes, resulting in resistance to rifampicin and quinolones, respectively [102]. The bacterial DNA gyrase B subunit (GyrB) may serve as a promising target for discovering and developing a new class of antibiotics [103]. It can bind to ATP in the ATPase domain and catalyze ATP hydrolysis, providing energy for DNA supercoiling [104]. When the GyrB inhibitor novobiocin was introduced into clinical use, antibiotics with the same mechanism of action could be considered promising therapies to fight bacterial infections [105, 106]. Methylation is another prevalent pathway of target modification and is considered to be a highly effective method in the development of resistance. Notably, through methylation of rRNA such as 23 S rRNA or 16 S rRNA methylation, bacteria have developed significant resistance by reducing or completely blocking antibiotics (such as penicillin) binding to the target [99]. Methylation of rRNA can be achieved by the action of certain enzymes [107]. Methyltransferases are the emerging group of target-modifying enzymes with key roles in modifying rRNA elements on the ribosome, thus resulting in the development of resistance to a variety of antibiotics, such as lincosamide, aminoglycoside, macrolide, streptogramin, and oxazolidinone. Recently, Tsai et al. addressed an important unresolved mechanism of AMR resulting from an RNA-modifying enzyme Cfr. The methylation of the rRNA residue A2503 on the large ribosomal subunit confers resistance to multiple ribosome-targeting antibiotics [108]. Other examples of methylation, including erythromycin resistance methylases (*erm*), render bacteria against some clinically important antibiotics such as lincosamides, macrolides, and streptogramin B [109]. A growing number of enzymes of increasing clinical importance have been identified to alter the molecular targets of antibiotics. These resistant factors emerged soon after the antibiotics started being used and are now widespread clinically and environmentally. Increasing exploration and understanding of the target-modifying enzymes-mediated resistance mechanisms provides opportunities to mentor the development of next-generation antibiotics capable of overcoming these emerging resistance strategies [110]. Future studies should be aimed at discovering antibiotic adjuvants for these identified enzymes.

### Target protection

A type of resistance mechanism does not fall into these basic categories and is not as clear and in-depth as other mechanisms in terms of clinical impact and mechanism understanding. One of these concerns is the phenomenon of a resistance mechanism called target protection [19]. Target protection involves a given resistance protein physically associated with an antibiotic target (or binding site) in the bacteria, known as “target protection proteins”, releasing the antibiotic from the developed resistance. One class of such proteins, widely distributed throughout gram-positive bacteria, are the AMR ATP-binding cassette proteins of the F-subtype capable of alleviating translational inhibition from antibiotics via binding the ribosome. Unlike common target modification mechanisms, the protein-to-protein interaction between the resistance protein (target protection protein) and the target only occurs once in principle. It leads to a chemical change of the latter. The target protection mechanism is not involved in permanent changes in the properties of the target. In contrast, sustained or repeated direct interactions between the target protection protein and the target are needed to confer resistance. Since the discovery of the first target protection mechanism regarding tetracycline resistance more than 30 years ago [111], it remains the only clearly documented example for quite a long time. Consequently, target protection is often considered nothing more than an unusual case among known mechanisms of bacterial resistance to antibiotics. It was thought to have limited impact in mediating clinically significant resistance, and descriptions of AMR mechanisms in previous works of literature often make little mention of it or cannot tell the differences between target protein and target modification mechanisms. Nevertheless, an increasing number of studies have shown that target protection plays a vital role in the development of clinically significant AMR, which is prevalent among different pathogenic bacteria [18]. Two proteins, including BceAB-type transporter and helicase-like protein (HelR) are explored here for the recently reported target protection mechanism.

#### BceAB-type transporter

**Fig. 5:**
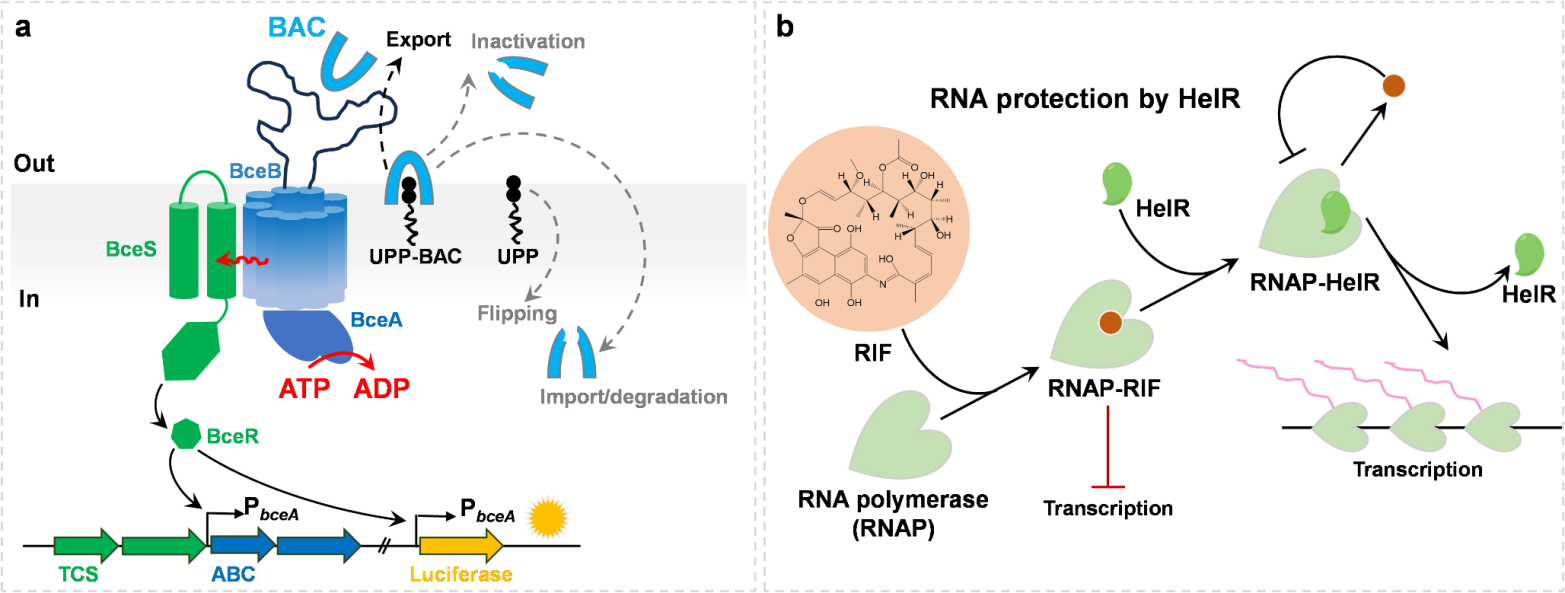
Target protection mechanism for developing resistance to antibiotics. (**a**) BceAB-BceRS transport system-mediated AMR against bacitracin (BAC) [117]. (**b**) HelR acts as a helicase-like protein for protecting RNA polymerase from rifamycin (RIF) antibiotics [118]

Antimicrobial peptides (AMPs) represent the most promising alternatives to antibiotics by targeting the bacterial cell wall and its core biosynthetic pathway (lipid II cycle), which are the most clinically important and significant antimicrobials, especially against gram-positive bacteria without the protective outer membrane. They are crucial for combatting the emerging wave of resistant bacteria via trapping lipid II cycle intermediates and preventing key enzymes from catalyzing the reaction efficiently [112]. However, bacteria can always develop an astonishing array of strategies against the antibiotic attack. Among a variety of known resistance mechanisms, the ATP-binding cassette transporters capable of removing AMPs from their action sites were considered a common strategy [113]. The BceAB-type transporters are a major group of these and have been identified from many environmental and pathogenic species [114]. BceAB mediates resistance against bacitracin, actagardine, mersacidin, and plectasin; bacitracin specifically binds the intermediate undecaprenyl pyrophosphate (UPP) of the lipid II cycle, thereby inhibiting its dephosphorylation, whereas the other AMPs bind lipid II itself [115]. After that, it was proposed that the BceAB transporter may act as a UPP flippase [116], which confers resistance by transporting UPP across the membrane to the cytoplasmic surface, thereby removing the cellular target for bacitracin instead of transporting bacitracin itself. More recently, Kobras et al. showed that the key determinant of BceAB transport activity was that bacitracin formed a complex with its cellular target UPP, but bacitracin or the lipid carrier alone [117] (Fig. 5a). It was then concluded that BceAB-type transporters could transiently release the cellular targets from the inhibitory control of the AMP and confer AMR through target protection of cell wall synthesis. Based on our knowledge, phage or mRNA display, and *de novo* synthesis technologies have been applied to design and produce novel AMPs, which have substantially contributed to the development of artificial AMPs. We anticipate these new AMPs will be good alternatives to current antimicrobials with potent antimicrobial activity and unique mechanisms.

#### Helicase-like protein HelR

By inhibiting RNA polymerase (RNAP), rifamycins exhibit a broad-spectrum bactericidal activity against various gram-positive and gram-negative pathogens. Although resistance to the rifamycin family of antibiotics in clinical isolates generally results from point mutations in the RNAP, many bacteria possess highly specific enzymes responsible for modifying and inactivating rifamycins. For controlling the expression of these enzymes, a helicase-like protein, namely HelR, was identified and employed in *Streptomyces venezuelae*, which conferred broad-scope resistance to rifamycin [118]. HelR can rescue transcription inhibition by displacing rifamycins from RNAP and forming a complex with RNAP, thus providing resistance via target protection (Fig. 5b). Similarly, HelR interacts with RNA polymerase to confer intrinsic rifamycin resistance in *Mycobacterium abscessus* [119]. HelRs are also widely distributed in *Actinobacteria*, thus providing a novel formidable challenge for the development of new rifamycin antibiotics. Understanding the resistance mechanism of HelR contributes to guiding the manufacturing of updated rifamycin analogues unsuitable for HelR, offering more effective rifamycin-led therapy for bacterial infections.

## Conclusions and future considerations

AMR has been recognized as an increasing threat to global human, animal, and environmental health, and the threat is accelerating. Meanwhile, since the 1980s, the discovery and development of new antibiotics have significantly lagged and are far behind the speed of AMR emergence. The occurrence of AMR is a natural ecological phenomenon in which bacteria have evolved over billions of years to resist the effects of antibacterial agents. Although bacterial resistance to antibiotics has been observed for a long time, it is only in recent years that we have gained some understanding of the biochemistry of how different antibiotics work and the mechanisms associated with the development of resistance. Advances in multi-omics, structural biology, molecular biology, and systems biology have dissected the accurate events in the development of resistance in many bacteria. They will continue to provide discoveries and expand our understanding. With a better understanding of host fitness and selection for resistance, we can accelerate the drug discovery process and develop new antimicrobial agents that can bypass or neutralize existing resistance mechanisms. A deep understanding of resistance development at genetic levels is a foundation for a variety of rapid diagnostic methods being developed, which are expected to guide initial antibiotic selection and reduce the use of ineffective antibiotics. Indeed, early identification of those naturally occurring resistance mechanisms and targets capable of accommodating numerous structural changes should lead to halting the development of drugs that may fail clinically because of resistance. Understanding and predicting how and when resistance occurs, and potential synergies with drug combinations can also help develop dosing regimens that minimize the occurrence and emergence of resistance to current and new antibiotics, allowing these antibiotics to exert the best effect. While restoring the efficacy of existing antibiotics via rational and reduced use is critical, new antibiotics or synergistic treatment combinations are urgently needed, and understanding resistance is an important prerequisite for these efforts. This is especially critical in the short term, as new antibiotics that are just being developed are unlikely to enter widespread clinical practice quickly. Despite acquiring significant knowledge about the resistance mechanisms and obtaining a series of response strategies, the challenge now facing this field is how best to utilize existing technology, information, and expertise to ensure that the influence of antibiotic resistance is fully considered when urgently developing next-generation antibiotics. For future studies, the following recommendations for controlling the dissemination of antibiotic resistance in the environment are considerable.

1. The CRISPR-Cas genome editing system has evolved as a powerful tool for identifying and understanding resistance mechanisms, limiting horizontal gene transfer, preventing the spread of antibiotic resistance, and developing novel treatments. Using the CRISPR-Cas system, the environmental antibiotic-resistant bacteria carrying mobile genetic elements can be precisely targeted and eliminated, potentiating the efficacy of antibiotics.
2. The past few years have witnessed rapid advances in the remarkable ability of artificial intelligence (AI). Innovations in AI provide new opportunities for environmental antibiotic resistance research. The development of high-performance computing power combined with the rise of diverse machine-learning algorithms significantly accelerates and expands our search for novel antibiotics with new chemical structures and mechanisms. It also offers the capacity to detect known and infer yet-unknown AMR mechanisms and predict future outbreaks of antibiotic-resistant infections.
3. Vaccines have been demonstrated to play an important role in fighting AMR. Because vaccines are intended for preventive use by inducing typically polyfunctional antibodies and/or cellular immune responses, they can reduce the number of infectious disease cases, thereby reducing the use of antibiotics and the emergence and spread of AMR.
4. Emerging technologies such as single-cell analysis and high-throughput droplet microfluidics analysis of antibiotic-resistant bacteria enable more cost-effective recognition and management of infectious disease and a better understanding of the resistance mechanism acquisition and loss in a complex environment.
5. Antibiotic resistance is a complex and multifaceted issue involving the transfer of bacteria and genes between humans, animals, and the environment. Interactions resulting from genes, competing evolutionary pathways, and external stressors within microbial populations can accelerate resistance evolution. Mathematical models of antibiotic use and resistance could be used to estimate the expected evolution in response to different treatments in different populations within and between microbial communities.
6. Most current research on antibiotic resistance is primarily conducted at a laboratory or pilot scale. The novel methods and techniques should be applied to real samples from the environment and tested on a large scale as soon as possible. Future research in AMR is expected to include a greater focus on the combination of identified resistance mechanisms and field application.

## Data Availability

No datasets were generated or analysed during the current study.
